# Invisible epidemics: ethics and asymptomatic infection

**DOI:** 10.1007/s40592-020-00123-z

**Published:** 2020-12-16

**Authors:** Euzebiusz Jamrozik, Michael J. Selgelid

**Affiliations:** 1 The Ethox Centre & Wellcome Centre for Ethics and the Humanities, Nuffield Department of Population Health, University of Oxford, Oxford, UK; 2 Monash Bioethics Centre, Monash University, Clayton, VIC Australia; 3 Royal Melbourne Hospital Department of Medicine, University of Melbourne, Parkville, VIC Australia

**Keywords:** Asymptomatic infection, Carrier, Microbial determinism, Antimicrobial resistance, Isolation, Quarantine

## Abstract

Interactions between microbes and human hosts can lead to a wide variety of possible outcomes including benefits to the host, asymptomatic infection, disease (which can be more or less severe), and/or death. Whether or not they themselves eventually develop disease, asymptomatic carriers can often transmit disease-causing pathogens to others. This phenomenon has a range of ethical implications for clinical medicine, public health, and infectious disease research. The implications of asymptomatic infection are especially significant in situations where, and/or to the extent that, the microbe in question is transmissible, potentially harmful, and/or untreatable. This article reviews the history and concept of asymptomatic infection, and relevant ethical issues associated with this phenomenon. It illustrates the role and ethical significance of asymptomatic infection in outbreaks, epidemics, and pandemics–including recent crises involving drug resistance, Zika, and Covid19. Serving as the Introduction to this Special Issue of *Monash Bioethics Review*, it also provides brief summaries of the other articles comprising this collection.

## Introduction

In November 2018, the Brocher Foundation hosted the first academic workshop on ethical issues associated with asymptomatic infection. Entitled “Invisible epidemics: ethics and interventions for asymptomatic carriers of infection”, this event included presentations by authors of the papers in this Special Issue of *Monash Bioethics Review*, among others. More recent developments such as the coronavirus disease 2019 (Covid19) pandemic have subsequently underscored the need for more scientific and ethical analyses of asymptomatic infection. This article reviews the history and concept of asymptomatic infection, and ethical issues associated with this phenomenon. It illustrates the role and ethical significance of asymptomatic infection in outbreaks, epidemics, and pandemics–including recent crises involving drug resistance, Zika, and Covid19. Serving as the Introduction to this Special Issue of *Monash Bioethics Review*, we also provide brief summaries of the other articles comprising this collection.

Asymptomatic infections constitute an important public health problem. This is because many people with asymptomatic infections later develop disease and—whether or not this occurs^1^—some asymptomatic infections can be transmitted to other people (who, in turn, might or might not eventually develop disease themselves). One of the most dramatic illustrations of the risks posed by asymptomatic carriers is the 2010–2017 cholera epidemic centred in Haiti. Cholera was (re)introduced to Haiti by one or more asymptomatic carriers of cholera bacteria among United Nations peacekeepers from Nepal who were sent to assist the country after a devastating earthquake (Frerichs et al. 2012). The ensuing epidemic resulted in over 500,000 cases and over 7000 deaths (Frerichs et al. 2012). More commonplace examples include drug-resistant bacteria, which are often carried and spread by healthy people, only a minority of whom develop disease (Jamrozik and Selgelid 2019). Although one might think that the proper role of medical intervention is to prioritise the treatment of disease (e.g., infections causing significant symptoms), controlling asymptomatic infection can be one way of preventing disease both among asymptomatic carriers and others.

For some infections, symptomatic individuals represent the tip of an epidemiological ‘iceberg’ which is composed primarily of asymptomatic carriers. According to WHO, “up to one third of the world’s population is estimated to be infected” with latent tuberculosis (TB); and “5–10% of those infected will develop active TB disease over the course of their lives” (World Health Organisation 2018). Those who do eventually develop active TB lung disease may then go on to infect other people.

Even more ubiquitous are the microbes that cause common bacterial diseases. The human body contains more bacterial cells than human cells (Sender et al. 2016), and many of these bacteria are potentially pathogenic in healthy people. Moreover, bacteria with lower pathogenic potential (i.e., those that rarely, if ever, cause disease in healthy people) nevertheless often cause disease in immunocompromised people—in whom, for example, even the apparently harmless organisms in yoghurt or probiotics can cause death (Salminen et al. 2004).

Many asymptomatic infections are, however, associated with benefits. For example, the presence of certain organisms in the body can inhibit the establishment of more harmful pathogens (Deasy et al. 2015), and microbial transplants are sometimes used as therapy to achieve such benefits (Bakken et al. 2011). Various microorganisms likewise play important roles in human physiology—including digestive processes. We expect that the future results of current microbiome research programs will elucidate numerous additional benefits—as well as risks—of asymptomatic infection.

## History

Dr Robert Koch was one of the founders of modern microbiology, and his work is particularly well known for a set of postulates (first published in 1890) linking microbes with the causation of infectious disease (Gradmann 2010). Though variously expressed, one of Koch’s initial postulates was that the microbe putatively responsible for a disease should be found in all people suffering from the disease, but not in healthy individuals (Gradmann 2010). Koch soon realised that this did not hold true in all cases, since many potentially pathogenic organisms are frequently found in healthy people. For example, Koch observed that asymptomatic carriers of cholera, typhoid, and malaria could spread these diseases to others, and he is credited for inventing the concept of the carrier state (i.e., in which healthy people asymptomatically carry an infection) (Gradmann 2010).

Public awareness of asymptomatic carriage of infection increased, especially in English-speaking countries, with media reporting of the case of Mary Mallon (known as “Typhoid Mary”) beginning in 1907. Mallon was a cook working in New York who, although showing no signs of typhoid disease herself, spread typhoid bacteria to many other people, resulting in several deaths (Brooks 1996; Soper 1939). For the general population, this revealed an important truth: that “persons, rather than things” (Soper 1939) were the source of many infectious diseases. Despite this Copernican revolution in public health (an epidemiological parallel of the microbiological revolution of germ theory), Mary Mallon and many others found it difficult to believe that healthy people could spread disease. Mallon repeatedly resisted public health restrictions and refused to believe she was infected or posed risks to others. She spent the latter years of her life living in public health confinement on North Brother Island, working as an assistant in the local infectious diseases laboratory (Soper 1939).

**Fig. 1:**
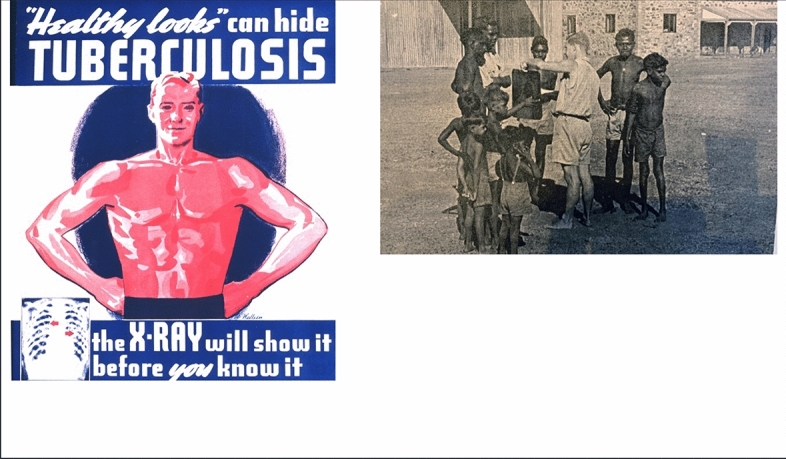
Public health tuberculosis screening programs in the USA (left) and Australia (right). Left: Image from the National Library of Medicine: http://resource.nlm.nih.gov/101451864. Right: Image courtesy Dr Alan King

Meanwhile, scientific advances permitted the demonstration of asymptomatic, or latent, forms of syphilis and tuberculosis. This created opportunities for mass screening (e.g., by blood tests and X-rays), treatment to prevent progression from asymptomatic infection to disease (in carriers), and (other) measures to prevent transmission to others (Fig. 1).

In more recent history, the global HIV epidemic led to a further increase in awareness of asymptomatic infection among the general public, scientists, and bioethicists. Discussions of HIV are not always generalisable to asymptomatic infection with other pathogens—for example, because HIV is associated with a long lag time between infection and disease (i.e., AIDS), its incidence was initially concentrated in identifiable social groups, it was initially untreatable, and the levels of fear and stigma related to the infection were (and perhaps remain) unusually high. However, HIV is in some respects a useful case study for more general issues surrounding asymptomatic infection, not only because HIV infection has been associated with considerable social stigma (like, for example, some drug-resistant infections today (Rump et al. 2017)), but also because the public health importance of asymptomatic infection is a function of the extent to which the pathogens in question are potentially deadly, transmissible, and/or untreatable. Although the development and use of antiretroviral drugs has reduced HIV mortality and transmission, all three features remain salient in contexts where (or for those for whom) HIV treatment is unavailable.

Globally rising tides of drug resistance make the problem of asymptomatic infection increasingly urgent—in part because certain highly resistant pathogens have become impossible to cure with existing antimicrobial drugs. For example, drug-resistant TB is a key threat to global public health, increasing numbers of people now asymptomatically carry multi-drug resistant TB (Nguyen et al. 2020), and some even carry “extensively” resistant strains which are virtually incurable. When, or if, such persons eventually develop active illness and thereby become contagious, the danger to others they might infect is significantly greater than would otherwise be the case.

All of the organisms included in the WHO List of Priority Resistant Bacteria can be carried asymptomatically. In each of these cases, the microbes are transmissible (even by healthy carriers^2^), the diseases associated with these microbes are potentially deadly, and treatment is becoming increasingly costly, difficult, and/or unsuccessful–especially in certain cases where pathogens have become resistant to many or *all* available antimicrobial drugs. Moreover, at the global level, many “second line” or “last line” antibiotics used for resistant infections are least available (often because of cost) in poor communities where resistance to cheaper and more effective “first line” drugs is higher and/or more common (Jamrozik and Selgelid 2020c). To the extent that asymptomatic infections are transmissible, deadly, and untreatable, there may be especially strong ethical justification for public health intervention (other things being equal).

## Conceptual matters

**Fig. 2:**
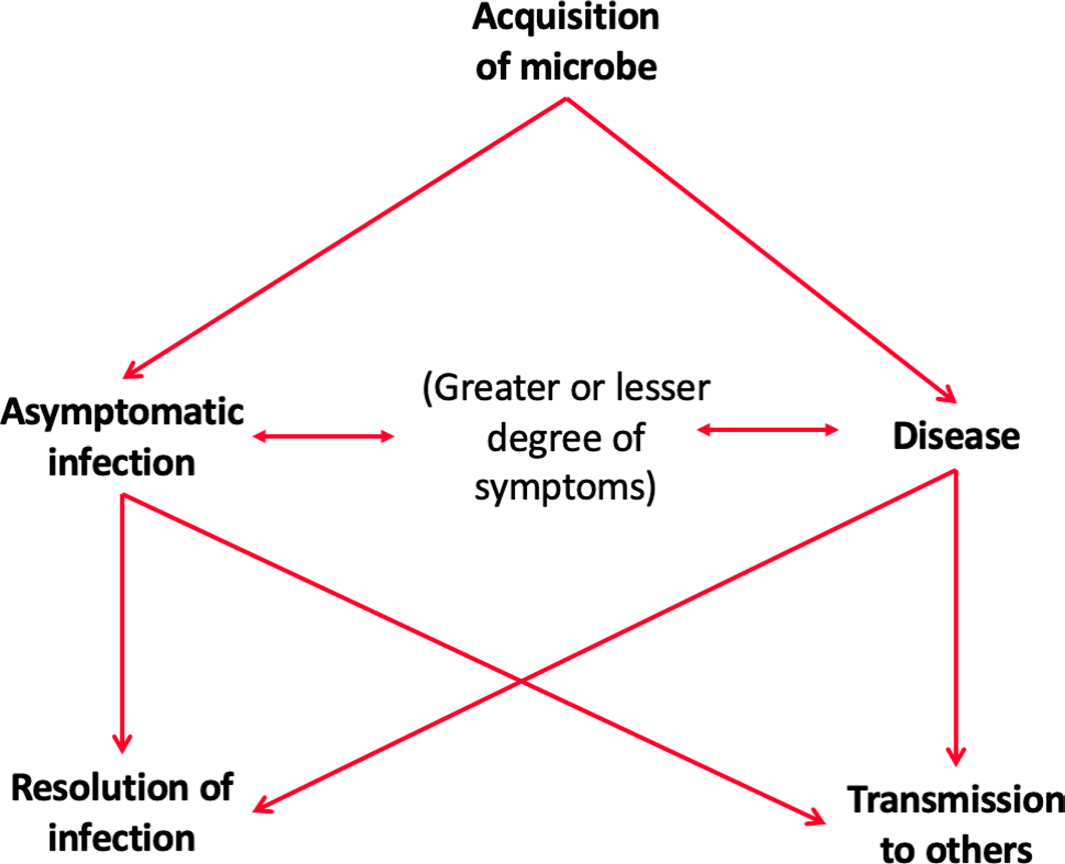
Potential consequences of infection with a microbe

The erroneous idea that the presence of a microbe determines whether someone will develop an infectious disease—which we call “microbial determinism” (Jamrozik and Selgelid 2019)—is closely analogous to the mistaken view of “genetic determinism”, according to which genotype determines phenotype. The presence of a given microbe in a given individual does not necessarily entail the development of an infectious disease; the microbe is merely one part of a complex set of processes (microbial, immune, cellular, social, etc.) which may result in various possible outcomes ranging from no symptoms to overt disease (which could be more or less severe) (See Fig. 2) (Casadevall and Pirofski 2020). In some cases, an infection resolves without ever causing symptoms. In other cases, an asymptomatic period (sometimes referred to as a period of “pre-symptomatic infection” or “the incubation period”) precedes more overt disease. Other more unusual patterns (depending on the microbe/pathogen) include relapsing symptomatic periods alternating with asymptomatic infection (as commonly occurs, for example, with herpes virus and vivax malaria) or an initial symptomatic infection followed by asymptomatic infection which may later develop into more sinister pathology (as commonly occurs with HIV, syphilis, and viral hepatitis). It is increasingly recognised that many transmissible longer-duration infections that often result in few or no symptoms initially can in some cases contribute to the risk of certain non-communicable diseases (e.g., viral hepatitis and cirrhosis or liver cancer, *H. pylori* and stomach cancer, human papillomavirus and cervical cancer, group A streptococcal infection and rheumatic heart disease, Chagas and cardiomyopathy, and infections associated with leukemias or lymphomas such as Epstein-Barr virus and human T-lymphotropic virus type 1, etc.) (Coates et al. 2020).

While some patterns of pathogenesis are commonly associated with “infection” by particular pathogens, the falsity of “microbial determinism” is partly reflected by the fact that outcomes so frequently vary from individual from individual—depending on other factors. Just like phenotype is caused by interaction between genes and environment (rather than being caused/determined by one or the other, or by genes alone) (Kaplan 2000) states of infection are determined by interactions between microbes and organisms (rather than being caused/determined by one or the other, or by microbes alone).

In some cases, standard characterisations of public health interventions should be revised in order to account for asymptomatic infection. For example, according to the American Centers for Disease Control and Prevention (CDC) ‘quarantine’ is typically defined as an intervention that “separates and restricts the movement of people who were *exposed* to a contagious disease *to see if they become sick*” whereas ‘isolation’ refers to an intervention that “separates *sick people with a contagious disease* from people who are not sick” (CDC 2020a, italics added) (See Box 1). Thus defined (assuming that ‘sick’ denotes individuals who are unwell, i.e., those with symptoms), neither of these interventions would adequately account for the public health importance of asymptomatic infection. Insofar as the goal of both quarantine and isolation is to reduce the transmission of infection, one would have thought that (1) the role of quarantine should be *to restrict the movement of an exposed person (or persons) until it can be determined* *whether they* *are* *infected with a transmissible pathogen (whether or not they become sick)* and (2) the role of isolation should be *to separate those infected with transmissible pathogens (whether or not they are sick) from others*—see Box 1.

**Table:** Box 1: Revising definitions of quarantine and isolation to account for asymptomatic infection

Standard definitions of quarantine and isolation:
*Quarantine* “The separation and restriction of movement of people who were exposed to a contagious disease to see if they become sick”
*Isolation* “The separation of sick people with a contagious disease from people who are not sick”
Revised definitions of quarantine and isolation accounting for asymptomatic infection:
*Quarantine* “The separation and restriction of movement of people who were exposed to transmissible pathogens to determine whether they are infected”
*Isolation* “The separation of those infected with transmissible pathogens from others”

Moreover, the CDC appears to use these terms differently (and along the lines we have suggested above) when referring to quarantine measures for Covid19 as those that help to “prevent spread of disease that can occur before a person knows they are sick or *if they are infected with the virus without feeling symptoms*” (CDC 2020b, italics added) and referring to isolation as aiming to “separate *people infected* … from people who are not infected” (CDC 2020c, italics added). This use of these terms aptly applies to asymptomatic cases and provides a better general characterisation of these measures than CDC’s more usual/standard definitions cited above—which (among other problems) seem to (in some ways) mistakenly assume the truth of “microbial determinism” (i.e., the idea that those infected with transmissible pathogens are, or will become, sick).

## Ethical issues

Asymptomatic infection has important ethical implications for clinical medicine, public health policy and practice, and infectious disease research. In clinical ethics, one relevant question relates to the conditions under which, if any, health practitioners should screen and/or treat individuals who may harbor asymptomatic infections. Answers to such questions will turn partly on the balance of benefits and harms (of screening or treatment) for individual patients (e.g., in some cases screening or treatment might actually entail a net expected harm for a particular patient). Clinical decision-making should arguably also turn on broader public health goals such as the cost-effectiveness of particular screening or treatment strategies (Krantz et al. 2004; Wilkinson et al. 2000) and the potential contribution that treatment might make to the prevalence of drug-resistant infections. It has been recognized, for example, that the latter consideration might entail limits on physicians’ duties to act in a particular patient’s best interests in order to protect public health (Wendler 2010; Oakley 2020). In some cases, treating a patient’s asymptomatic infection with antimicrobials (e.g., if the infection is likely to otherwise develop into a life-threatening disease) might involve a significant benefit that outweighs the public health risk (of promoting drug resistance); in others, treatment might entail a net risk for the patient and/or for public health. In addition, under certain circumstances (e.g., when risks to third parties and/or public health are sufficiently high) physicians may be justifiably required to report an asymptomatic infection to public health authorities (an exception to usual assurances of patient confidentiality) (Francis and Francis 2020).

In public health ethics, asymptomatic infection has implications for a range of questions for policy and practice (Rump et al. 2017, 2020, 2018; Voo and Lederman 2020; Holm 2020; Douglas 2019; Nijsingh et al. 2020). A relatively unique set of questions relates to the extent to which the ethical acceptability of an individually burdensome control measure is contingent on the degree to which the person subject to the measure experiences symptoms. Answers to such general questions can help to inform policy decision-making regarding the justifiability of interventions such as screening, reporting/notification, monitoring, isolation, quarantine, and/or travel bans targeting (potential) carriers (See Box 2). Similar considerations may also inform evaluations of the conditions under which it would be justifiable *to enforce or mandate* particular measures (Douglas 2019), and those under which asymptomatic individuals should be compensated for the burdens imposed by public health intervention (See Box 2) (Holm 2020).

Evaluations of the appropriateness of public health responses will in some cases turn on empirical matters including epidemiological data (e.g., related to the proportion of infected individuals demonstrating symptoms, the duration and reversibility of asymptomatic carriage, the degree to which cases of disease are due to transmission from asymptomatic individuals and/or the degree to which symptoms are correlated with risk of transmission) as well as information regarding the proposed public health intervention (e.g., related to the accuracy of diagnostic testing and the likely effectiveness of a proposed treatment, or other measures, in terms of reducing disease among carriers and/or others).

Asymptomatic infection also serves as an important case study for more general debates in public health ethics such as those regarding the duty not to infect others (Harris and Holm 1995; Verweij 2005; Selgelid 2009). While many people might find it intuitive that a person who *knows* she is infected with a transmissible pathogen has strong moral reasons to take precautionary measures to reduce the risk of infecting others (Harris and Holm 1995), the situation with respect to asymptomatic infections and/or people who do not know whether they are infected might be more complex—or lead to more far-reaching and/or demanding duties (perhaps overdemanding duties if appropriate limits thereof are not recognised) (Verweij 2005; Selgelid 2009). On one plausible view, the strength of one’s duty to avoid infecting others should be proportional to the probability that one is infected and the risks that such an infection would impose on others, and one’s knowledge of such risks (Selgelid 2009). Such considerations are often highly context-dependent because, for example, in some cases symptoms (such as a cough) might increase the risk of transmission to others, whereas in other cases even entirely asymptomatic infections might entail significant transmission risks (Jamrozik and Selgelid 2019).

**Table:** Box 2: Examples of ethical policy questions related to asymptomatic infection

Under what conditions, if any, should public health agencies:
– Screen apparently healthy people for infectious diseases?
– Make carriage reportable/notifiable?
– Monitor asymptomatic carriers?
– Isolate carriers or quarantine their contacts?
– Make public health measures for carriers mandatory?
– Implement travel bans for carriers?
– Compensate carriers subjected to public health measures?

In research ethics, asymptomatic infection might also raise a range of ethical issues, for example, regarding appropriate policies regarding the management of (1) infections that are incidentally diagnosed as a result of research participation (similar to incidental findings in human genetics research) (Magiorkinis et al. 2019) or (2) infections intentionally identified during microbiome research (McGuire et al. 2012). The potential for such findings might have implications regarding the expected benefits and risks of relevant research (including both risks to participants and risks to third parties, who could potentially be infected by participants identified as carriers), as well as for consent and follow-up of participants. More generally, infectious disease research often involves potential risks to third parties (who might be infected by research participants) whether or not participants who carry an infection develop symptoms, and researchers might have particularly strong duties to mitigate such risks in certain types of research—such as human challenge studies, which involve intentional infection of research participants (Shah et al. 2018; Eyal et al. 2018; Jamrozik and Selgelid 2020a). It is noteworthy that asymptomatic infection is sometimes an explicit consideration in the design of vaccine trials, which sometimes (including during epidemics, discussed below) require detection of asymptomatic infection in order to determine vaccine efficacy (Kahn et al. 2019).

## Implications for outbreaks, epidemics, and pandemics

Asymptomatic infection was recognised to be a significant factor in the 2015–2016 Zika virus epidemic, particularly because many of those who were infected—including some women who acquired infection during pregnancy and gave birth to children severely affected by congenital Zika syndrome—showed few or no symptoms (Jamrozik and Selgelid 2018). Although less well recognised, transmission of asymptomatic Middle Eastern Respiratory Syndrome (MERS) coronavirus infection (perhaps both camel-human and human–human transmission) may play an important role in the epidemiology of MERS—which is all the more remarkable because people who develop *symptomatic* MERS infection have a high fatality risk of around 35% (Grant et al. 2019). Asymptomatic infection has also been reported for viruses closely related to the coronavirus that caused the earlier severe acute respiratory syndrome (SARS) epidemic. In one study from 2003, around 40% of Chinese wild animal traders had serological evidence of having been exposed to coronaviruses that closely resembled SARS-coronavirus, raising questions about whether people in high risk occupations should be screened for asymptomatic infection to detect potential “spillover” events of pathogens with epidemic potential (Guan et al. 2003). We initiated the November 2018 Brocher Foundation workshop upon which this Special Issue is based partly in light of the growing awareness of such cases of asymptomatic infection—and their ethical implications for policy and practice.

Since that time general awareness of asymptomatic infection has skyrocketed in light of its role in the coronavirus disease 2019 (Covid19) pandemic, in virtue of which the term ‘asymptomatic infection’ has become highly familiar to ordinary members of the general public. Early data, which were later widely confirmed, suggested that asymptomatic transmission of Covid19 occurs both in cases where the individual transmitting the virus goes on to develop symptoms later (i.e., they were “pre-symptomatic” at the time of transmission) and in cases where they never develop symptoms (Hu et al. 2020). Asymptomatic individuals can, under certain conditions, transmit to large numbers of other people (e.g., one person was shown to infect 71 others) (Liu et al. 2020). The overall degree to which asymptomatic transmission contributes to local Covid19 epidemics likely varies in different contexts and has not always been well-characterised (in part because of the difficulties of identifying all asymptomatic infections during an epidemic). In any case, asymptomatic transmission of Covid19 raises a number of ethical issues similar to those discussed above, including those related to the justification of public health interventions such as screening and isolation for asymptomatic cases.

Asymptomatic infection therefore has important implications during epidemics. First, early assessments of the risk of an epidemic disease (e.g., case fatality risk—the proportion of reported cases resulting in death) are frequently biased towards overestimation because the earliest notified cases more often fall towards the severe end of the spectrum of disease. Although asymptomatic cases of infection are (initially) harder to detect, the eventual inclusion of such cases in “the denominator” (in the determination of “infection fatality risk”—i.e., the proportion of infections that result in death) often demonstrates that a given kind of infection is less risky than was suggested by early estimates (Lipsitch et al. 2015; Verity et al. 2019). Second, when, or insofar as, asymptomatic (or mild) infection confers some degree of immunity, this may prove beneficial in the protection individuals against future infection (which might otherwise be more severe, for example, because the probability of severe Covid19 increases with age, meaning that individuals are in some cases arguably better off being infected earlier in life—at least if, or insofar as, acquired immunity proves to be long-lasting). Moreover, insofar as such immunity prevents individuals from infecting others, it may provide wider benefits by contributing to herd immunity.

Third, just as considerations related to asymptomatic infection may influence the ethical acceptability of ordinary public health measures, similar considerations may apply to the augmented public health measures proposed to control an epidemic. Certain additional public health measures (targeting apparently healthy individuals) may be justifiable to the extent that asymptomatic infections are likely to be highly transmissible, deadly, and untreatable, whereas other measures may entail restrictions or harms that are disproportionate to the benefits they purportedly provide. Evaluations of the ethical acceptability of such additional public health measures during an epidemic may partly depend on the ease of detection of, and risk imposed by, asymptomatically infected individuals (Jamrozik and Selgelid 2018). To the extent that failure to detect asymptomatic infection undermines the purported public health benefits of a public health interventions, such interventions will likely be cost-ineffective and difficult to justify on ethical grounds, especially if they also involve liberty infringements or other burdens on individuals.

Fourth, asymptomatic infection will often influence the design of research and/or the interpretation of research results during epidemics. For example, vaccine field trials (in which participants are given an experimental vaccine or placebo and then monitored for the infection in their daily lives) need to be particularly carefully designed and conducted where failure to account for asymptomatic infection would undermine the ability of such trials to produce accurate results regarding vaccine effectiveness (Kahn et al. 2019). Moreover, certain types of research, if properly designed, may be able to elucidate the transmission risks posed by asymptomatic individuals and thus better inform public health policies (Jamrozik and Selgelid 2020b).

## Contributions in this special issue

This Special Issue on Ethics and Asymptomatic Infection includes five papers on specific topics. Thomas Douglas considers the justification of burdensome infection control measures in the context of infectious diseases via analogies to the use of “crime control” measures in criminal justice, noting that both types of measures invoke the prevention of third-party risk as a justification for infringements on individual rights, and that these measures are often mandatory (Douglas 2019). *Inter alia,* Douglas argues that mandatory measures might be less morally problematic in cases where (i) voluntary measures have been attempted and failed, (ii) the epidemiology of the infectious disease does not track prior disadvantage and/or is not associated with stigmatised behaviour or groups, (iii) those subjected to control measures are not thereby exposed to greater risks of infection (e.g., this might be an important consideration when quarantine of potentially infected individuals together would entail a risk that those who are already infected will infect others who would not have been infected had they not been quarantined), (iv) measures are targeted in a fine-grained rather than overly inclusive way (e.g., so that the burdens of measures are proportionate to the risks created by infected individuals, not merely the risks inferred from their membership of a particular social group), and (v) individuals burdened by control measures are appropriately compensated (Douglas 2019).

Søren Holm proposes a general framework for compensation in the context of public health measures for infectious diseases (Holm 2020). He argues that society has a strong *prima facie* obligation not only to compensate individuals burdened by such measures, but also to ensure that such burdens are minimised. Holm advocates “no-fault compensation” that is easily accessible, efficient, and provides adequate reparations to all those who have valid claims (i.e., to all who are burdened/harmed as a result of a public health measures whose benefits are widely shared). In addition to such compensation being owed to individuals because they are burdened by public health measures, an additional consideration in favour of compensation provision is that this may increase compliance with public health measures (while uncompensated burdens might result in perverse incentives for individuals to refrain from compliance).

The remaining three papers focus on drug-resistant infections. Babette Rump and colleagues provide an illuminating discussion of the experience of asymptomatic carriers of resistant pathogens, complementing other work by their group in this area and adding to other analyses of solidarity in the context of drug resistance (Rump et al. 2020; Byskov et al. 2020; Holm and Ploug 2020). Rump et al. argue that the design of infection control measures should be reframed according to a principle of solidarity that entails asking how carriers can be cared for without imposing unacceptable risks on others (Rump et al. 2020). The authors argue that a solidarity-based approach could include elevating baseline levels of universal precautions where feasible (i.e., considering all patients as potential carriers of resistant pathogens). Moreover, they suggest that “zero-risk tolerance” infection control policies should in at least some cases be revised, in part because such policies often entail an unreasonable level of burdens for some carriers (Rump et al. 2020; Millar 2012). Rump et al. additionally support appropriate compensation for carriers who are burdened by public health measures, including coverage of the costs of any medical treatment as well as lost income, where relevant (Rump et al. 2020).

Teck Chuan Voo and Zohar Lederman focus on justice considerations related to the ethical acceptability of control measures for methicillin-resistant *S. aureus* (MRSA) infection in healthcare settings (Voo and Lederman 2020). Like Rump et al. they note a tension between universal (or horizontal) infection control policies and targeted (or vertical) policies aimed at a particular pathogen such as MRSA. The latter often involve intensive surveillance (to detect carriers) and intervention, including among asymptomatic carriers, to prevent the spread of infection. Voo and Lederman note that intensive targeted policies are widely endorsed in outbreak situations (where there is a short-term rapid increase in the incidence of MRSA), but are more controversial when implemented as routine practice (Voo and Lederman 2020). Those who oppose such practices argue that they entail various forms of injustice for carriers of MRSA, at least in some cases. Voo and Lederman agree that current practices often are unjust but argue for a shift in the frame of the debate from healthcare settings to the level of public health—where public health ethics approaches are well-equipped to help determine appropriate policies. Rather than abandon MRSA-targeted strategies entirely, they recommend more high-quality research aimed at establishing highly (cost-)effective strategies as well as interventions that would reduce individual burdens and costs for carriers. With better evidence in hand, according to Voo and Lederman, there would be strong justification for adequate funding to ensure the consistent application of such policies.

Niels Nijsingh et al. analyse the ethical acceptability of screening programs for asymptomatic carriage of multi-drug-resistant gram-negative (MDRGN) bacterial infections in low prevalence settings (i.e., high-income countries) and compare these with similar programs for MRSA (Nijsingh et al. 2020). Resistant gram-negative bacteria include key WHO “priority pathogens” such as carbapenem-resistant Enterobacteriaceae which are widely considered important global public health threats, already cause a large global burden of disease, and yet are most frequently carried asymptomatically in the digestive tract of otherwise healthy people (Jamrozik and Selgelid 2019). Nijsingh et al. argue that routine hospital screening for asymptomatic infection in low prevalence settings is relatively disproportionate (as compared to MRSA screening policies) and advocate more targeted strategies aimed at preventing *symptomatic* infection and hospital outbreaks. For example, they suggest limiting screening to cases where the rates of true positive tests and/or resistant *disease* in the screened individual are likely to be higher and/or where the wider consequences of an outbreak of MDRGN disease in particular hospital units are likely to be severe (e.g., where there is a high prevalence of immunosuppression). In such cases, they argue, the restrictions and/or harms entailed by screening programmes may be more proportionate. *Inter alia*, Nijsingh et al. also advocate that stigma be reduced by education (of healthcare workers, patients, and members of the public) and by less restrictive, and thus more equitable, measures for carriers of MDRGN bacteria. Consistent with several other papers in this Special Issue, the authors support consideration of compensation for carriers to offset any excess burdens imposed by control measures. Although Nijsingh et al. focus on low-prevalence high-income settings, it is fruitful to consider the other end of the spectrum; for example, a study in Malaysian hospitals found that around 50% of patients carried MDRGN bacteria and the authors of that study advocated abandonment of universal screening partly because of the infeasibly high costs involved (Zaidah et al. 2017).

## Conclusions

Asymptomatic infection is associated with a range of risks and benefits for asymptomatic carriers and for others to whom they might transmit infection. Such infections have frequently played a role in the development and persistence of outbreaks, epidemics, and pandemics, including rising global incidence of drug-resistant infections. The public health importance of asymptomatic infection—and thus the justifiability of potentially infringing response measures—is a function of the extent that the microbe in question is transmissible, potentially harmful, and/or untreatable. Such infections have ethical implications for clinical medicine, public health, and infectious diseases research. Among other things, policy deliberations regarding infectious diseases should involve ethical analysis that weighs the restrictions and/or burdens against the potential public health benefits of potential interventions.

## Footnotes

1 In the context of TB, transmission from carriers usually only occurs when they transition from a latent/asymptomatic phase to active illness. In a great many cases of other potential pathogens, however, transmission occurs from asymptomatic (apparently healthy) carriers to others.

2 See footnote 1.

## References

[CR2] Bakken JS, Borody T, Brandt LJ, Brill JV, Demarco DC, Franzos MA (2011). Treating Clostridium difficile infection with fecal microbiota transplantation. Clinical Gastroenterology and Hepatology..

[CR3] Brooks J (1996). The sad and tragic life of Typhoid Mary. CMAJ: Canadian Medical Association Journal.

[CR4] Byskov MF, Rump BO, Verweij M, Jamrozik E, Selgelid MJ (2020). Conceptualizing the impact of MDRO control measures directed at carriers: A capability approach. Ethics and drug resistance: Collective responsibility for global. Public Health public health ethics analysis.

[CR5] Casadevall A, Pirofski L (2000). Host-pathogen interactions: Basic concepts of microbial commensalism, colonization, infection, and disease. Infection and Immunity.

[CR51] Centers for Disease Control and Prevention. 2020a. About Quarantine and Isolation. https://www.cdc.gov/quarantine/quarantineisolation.html. Accessed 9 Dec 2020.

[CR52] Centers for Disease Control and Prevention. 2020b. When to Quarantine: Stay home if you might have been exposed to COVID-19. https://www.cdc.gov/coronavirus/2019-ncov/if-you-are-sick/quarantine.html. Accessed 9 Dec 2020.

[CR53] Centers for Disease Control and Prevention. 2020c. Isolate If You Are Sick: Separate yourself from others if you have COVID-19. https://www.cdc.gov/coronavirus/2019-ncov/if-you-are-sick/isolation.html. Accessed 9 Dec 2020.

[CR6] Coates MM (2020). Burden of non-communicable diseases from infectious causes in 2017: A modelling study. Lancet Global Health.

[CR7] Deasy AM, Guccione E, Dale AP, Andrews N, Evans CM, Bennett JS (2015). Nasal inoculation of the commensal Neisseria lactamica inhibits carriage of Neisseria meningitidis by young adults: A controlled human infection study. Clinical Infectious Diseases.

[CR8] Douglas, T. 2019. Infection control for third-party benefit: Lessons from criminal justice. *Monash Bioethics Review*. 10.1007/s40592-019-00103-y PMC7749867 31832972

[CR9] Eyal N, Lipsitch M, Bärnighausen T, Wikler D (2018). Risk to study nonparticipants: A procedural approach. Proceedings of the National Academy of Sciences.

[CR10] Francis JG, Francis LP, Jamrozik E, Selgelid MJ (2020). Fairness in the use of information about carriers of resistant infections. Ethics and drug resistance: Collective responsibility for global public health. Public health ethics analysis.

[CR11] Frerichs RR, Keim PS, Barrais R, Piarroux R (2012). Nepalese origin of cholera epidemic in Haiti. Clinical Microbiology and Infection.

[CR13] Gradmann, C. 2010. Robert Koch and the invention of the carrier state: Tropical medicine, veterinary infections and epidemiology around 1900. *Studies in History and Philosophy of Science Part C: Studies in History and Philosophy of Biological and Biomedical Sciences* 41 (3): 232–240. 10.1016/j.shpsc.2010.04.012 20934644

[CR14] Grant R, Malik MR, Elkholy A, Van Kerkhove MD (2019). A review of asymptomatic and subclinical middle east respiratory syndrome coronavirus infections. Epidemiologic Reviews.

[CR15] Guan Y, Zheng B, He Y, Liu X, Zhuang Z, Cheung C (2003). Isolation and characterization of viruses related to the SARS coronavirus from animals in southern China. Science.

[CR16] Harris J, Holm S (1995). Is there a moral obligation not to infect others?. British Medical Journal.

[CR17] Holm, S. 2010. A general approach to compensation for losses incurred due to public health interventions in the infectious disease context. *Monash Bioethics Review* 1–15. 10.1007/s40592-020-00104-2 PMC7095444 32130682

[CR18] Holm S, Ploug T, Jamrozik E, Selgelid MJ (2020). Solidarity and antimicrobial resistance. Ethics and drug resistance: Collective responsibility for global. Public health public health ethics analysis.

[CR19] Hu Z, Song C, Xu C, Jin G, Chen Y, Xu X (2020). Clinical characteristics of 24 asymptomatic infections with COVID-19 screened among close contacts in Nanjing, China. Science China Life Sciences.

[CR20] Jamrozik E, Selgelid MJ (2018). Ethics, health policy, and Zika: From emergency to global epidemic?. Journal of Medical Ethics.

[CR21] Jamrozik E, Selgelid MJ (2019). Surveillance and control of asymptomatic carriers of drug-resistant bacteria. Bioethics.

[CR22] Jamrozik E, Selgelid MJ (2020). Human challenge studies in endemic settings: Ethical and regulatory issues.

[CR23] Jamrozik, E., and M.J. Selgelid. 2020b. COVID-19 human challenge studies: Ethical issues. *The Lancet Infectious Diseases*. 10.1016/S1473-3099(20)30438-2 PMC7259898 32479747

[CR24] Jamrozik E, Selgelid MJ, Jamrozik E, Selgelid MJ (2020). Drug-resistant infection: causes, consequences, and responses. Ethics and drug resistance: Collective Responsibility for global public health. Public health ethics analysis.

[CR25] Kahn R, Hitchings M, Wang R, Bellan SE, Lipsitch M (2019). Analyzing vaccine trials in epidemics with mild and asymptomatic infection. American Journal of Epidemiology.

[CR26] Kaplan JM (2000). The limits and lies of human genetic research: Dangers for social policy.

[CR27] Krantz I, Löwhagen G-B, Ahlberg BM, Nilstun T (2004). Ethics of screening for asymptomatic herpes virus type 2 infection. BMJ.

[CR28] Lipsitch M, Donnelly CA, Fraser C, Blake IM, Cori A, Dorigatti I (2015). Potential biases in estimating absolute and relative case-fatality risks during outbreaks. PLoS Neglected Tropical Diseases.

[CR29] Liu J, Huang J, Xiang D (2020). Large SARS-CoV-2 outbreak caused by asymptomatic traveler, China. Emerging Infectious Diseases.

[CR30] Magiorkinis, G., P.C. Matthews, S.E. Wallace, K. Jeffery, K. Dunbar, R. Tedder, et al. 2019. Potential for diagnosis of infectious disease from the 100,000 Genomes Project Metagenomic Dataset: Recommendations for reporting results. *Wellcome Open Research* 4. 10.12688/wellcomeopenres.15499.1 PMC6993825 32055707

[CR31] McGuire AL, Achenbaum LS, Whitney SN, Slashinski MJ, Versalovic J, Keitel WA (2012). Perspectives on human microbiome research ethics. Journal of Empirical Research on Human Research Ethics.

[CR32] Millar M (2012). ‘Zero tolerance’of avoidable infection in the English National Health Service: Avoiding the redistribution of burdens. Public Health Ethics.

[CR33] Nguyen B, Fox GJ, Mason PH, Denholm JT, Jamrozik E, Selgelid MJ (2020). Preventive therapy for multidrug resistant tuberculosis: An ethical imperative with ethical barriers to implementation?. Ethics and drug resistance: Collective responsibility for global public health. Public health ethics analysis.

[CR34] Nijsingh, N., C. Munthe, A. Lindblom, and C. Åhrén. 2020. Screening for multi‑drug‑resistant Gram‑negative bacteria: What is effective and justifiable? *Monash Bioethics Review*. 10.1007/s40592-020-00113-1 PMC7749868 32356217

[CR35] Oakley J, Jamrozik E, Selgelid MJ (2020). The virtuous physician and antimicrobial prescribing policy and practice. Ethics and drug resistance: Collective responsibility for global public health. Public health ethics analysis.

[CR36] Rump B, De Boer M, Reis R, Wassenberg M, Van Steenbergen J (2017). Signs of stigma and poor mental health among carriers of MRSA. Journal of Hospital Infection.

[CR37] Rump B, Timen A, Hulscher M, Verweij M (2018). Ethics of infection control measures for carriers of antimicrobial drug–Resistant organisms. Emerging Infectious Diseases.

[CR38] Rump, B., A. Timen, M. Hulscher, and M. Verweij. 2020. Infection control measures in times of antimicrobial resistance—A matter of solidarity. *Monash Bioethics Review* 139. 10.1007/s40592-020-00119-9 PMC7648233 33159651

[CR39] Salminen MK, Rautelin H, Tynkkynen S, Poussa T, Saxelin M, Valtonen V (2004). Lactobacillus bacteremia, clinical significance, and patient outcome, with special focus on probiotic L. rhamnosus GG. Clinical Infectious Diseases.

[CR40] Selgelid MJ (2009). Pandethics. Public Health.

[CR41] Sender R, Fuchs S, Milo R (2016). Revised estimates for the number of human and bacteria cells in the body. PLoS Biology.

[CR42] Shah SK, Kimmelman J, Lyerly AD, Lynch HF, Miller FG, Palacios R (2018). Bystander risk, social value, and ethics of human research. Science.

[CR43] Soper GA (1939). The curious career of Typhoid Mary. Bulletin of the New York Academy of Medicine.

[CR44] Verity, R., L.C. Okell, I. Dorigatti, P. Winskill, C. Whittaker, N. Imai, et al. 2020. Estimates of the severity of coronavirus disease 2019: A model-based analysis. *The Lancet Infectious Diseases*. 10.1016/S1473-3099(20)30243-7 PMC7158570 32240634

[CR45] Verweij M (2005). Obligatory precautions against infection. Bioethics.

[CR46] Voo, T.C., and Z. Lederman. 2010. Justice in control of methicillin-resistant Staphylococcus aureus transmission: A fair question to ask? *Monash Bioethics Review* 1–16. 10.1007/s40592-020-00109-x 32285336

[CR47] Wendler D (2010). Are physicians obligated always to act in the patient's best interests?. Journal of Medical Ethics.

[CR48] Wilkinson D, Barton S, Cowan F (2000). HSV-2 specific serology should not be offered routinely to antenatal patients. Reviews in Medical Virology.

[CR1] World Health Organisation. 2018. Latent tuberculosis infection: Updated and consolidated guidelines for programmatic management. 30277688

[CR49] Zaidah AR, Mohammad NI, Suraiya S, Harun A (2017). High burden of Carbapenem-resistant Enterobacteriaceae (CRE) fecal carriage at a teaching hospital: Cost-effectiveness of screening in low-resource setting. Antimicrobial Resistance & Infection Control.

